# Benign Lichenoid Keratosis of the Breast: Clinicopathologic Correlation of a Rare Presentation

**DOI:** 10.7759/cureus.99029

**Published:** 2025-12-12

**Authors:** Shaikhah Alenezi, Alsadat Mosbeh, Abeer Albazali

**Affiliations:** 1 Dermatology, Farwaniya Hospital, Kuwait City, KWT; 2 Dermatology/Dermatopathology, Faculty of Medicine, Al-Azhar University, Cairo, EGY

**Keywords:** benign lichenoid keratosis, dermatology, dermatopathology, lichen planus-like keratosis, pigmented macule

## Abstract

Benign lichenoid keratosis (BLK), also known as lichen planus-like keratosis, is a regressive inflammatory lesion that often mimics melanocytic or inflammatory dermatoses. It typically arises in middle-aged to elderly individuals, most commonly on sun-exposed skin, and histologically represents a lichenoid inflammatory response within a pre-existing epidermal lesion such as solar lentigo or seborrheic keratosis. In the present paper, we report a rare case of BLK occurring on the breast of a 53-year-old woman who presented with a solitary, asymptomatic pigmented lesion on the right breast. Clinical examination revealed a well-defined, flat brown macule measuring approximately 0.5 cm, with no surface changes or regional lymphadenopathy. Histopathological examination revealed compact hyperkeratosis, basal vacuolar degeneration, a dense band-like lymphocytic infiltrate, apoptotic keratinocytes, and pigment incontinence with numerous dermal melanophages, features characteristic of BLK. The patient's lesion was completely excised with no recurrence at follow-up. This case highlights the diagnostic challenge of BLK at atypical, non-sun-exposed sites and underscores the importance of clinicopathologic correlation and histopathologic confirmation to prevent misdiagnosis and unnecessary treatment.

## Introduction

Benign lichenoid keratosis (BLK), also referred to as lichen planus-like keratosis (LPLK), is an acquired, regressive inflammatory lesion that typically develops within a pre-existing epidermal proliferation such as solar lentigo or seborrheic keratosis [1,2]. It most often affects middle-aged and older adults and shows a predilection for chronically sun-exposed sites, including the upper trunk, shoulders, and extremities [3]. Clinically, BLK presents as a solitary, flat, or slightly elevated papule or macule with colors ranging from erythematous to violaceous or brown, depending on the stage of regression [4]. These variable morphologic stages frequently overlap with the clinical appearance of other pigmented or inflammatory dermatoses, including lichen planus, seborrheic keratosis, actinic keratosis, or early melanoma, making clinical diagnosis challenging [5].

Although the exact pathogenesis of BLK remains incompletely understood, ultraviolet (UV) radiation, epidermal aging, and immune-mediated injury appear to play central roles [1,2]. UV-induced keratinocyte damage is believed to trigger a localized lichenoid inflammatory response, leading to basal cell vacuolar degeneration, pigment incontinence, and a characteristic band-like lymphocytic infiltrate at the dermoepidermal junction [2,5]. Histopathologic evaluation, therefore, remains essential in confirming the diagnosis and distinguishing BLK from melanocytic neoplasms, as features such as compact hyperkeratosis, basal cell degeneration, Civatte bodies, and a dense lichenoid infiltrate are characteristic findings [1,6].

While BLK is relatively common on photoexposed skin, its occurrence on non-sun-exposed sites, such as the breast, is exceptionally rare [4,7]. Pigmented lesions arising in this location often raise greater clinical concern because they may mimic melanoma or other neoplastic entities, increasing the likelihood of unnecessary excision or patient anxiety [5,7]. Recognizing BLK in atypical anatomical sites requires careful clinicopathologic correlation [2]. Recent clinicopathologic studies underscore the diagnostic variability of BLK and highlight the importance of integrating clinical appearance, histopathologic features, and, when available, dermoscopic patterns to ensure accurate diagnosis and avoid overtreatment [8].

In this paper, we present a rare case of BLK localized to the breast of a 53-year-old woman. We highlight its clinicopathologic characteristics and review current literature to enhance the recognition of this entity in unusual locations.

## Case presentation

**Figure 1:**
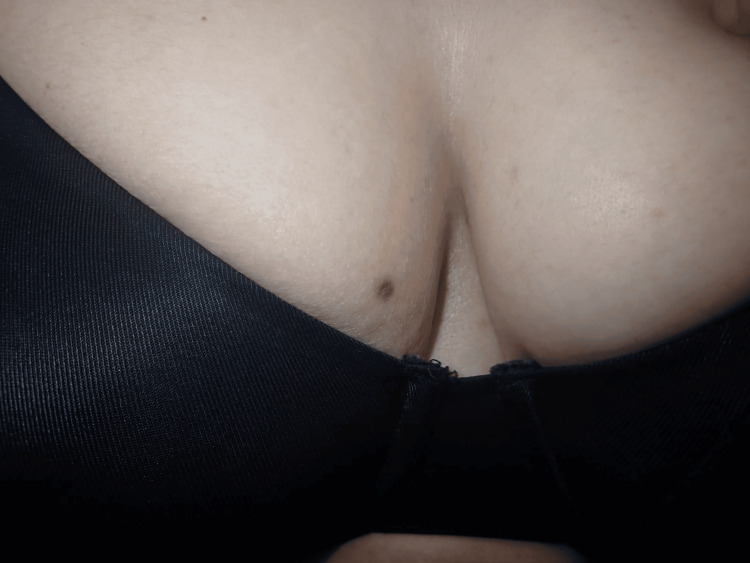
Clinical photograph showing a solitary, well-defined brown macule on the middle quadrant of the right breast

A 53-year-old woman with no significant past dermatologic history presented to the dermatology clinic with concern regarding a solitary pigmented macule on her right breast (Figure 1), which she had first noticed eight months earlier. The lesion was asymptomatic, with no associated pruritus, pain, bleeding, or change in size. She denied any history of trauma, excessive sun exposure to the area, or previous cutaneous malignancy. There was no relevant family history of dermatologic disease.

Clinical examination revealed a well-defined, flat brown macule measuring approximately 0.5×0.5×0.6 cm located on the middle inner quadrant of the right breast (Figure 1). The surface appeared smooth without scale, crusting, or ulceration. The surrounding skin was unremarkable, and no axillary or regional lymphadenopathy was detected.

Given the solitary nature and persistent pigmentation, an excisional biopsy was performed under local anesthesia for definitive diagnosis. The tissue specimen was fixed in formalin and processed for routine hematoxylin and eosin (H&E) staining.

**Figure 2:**
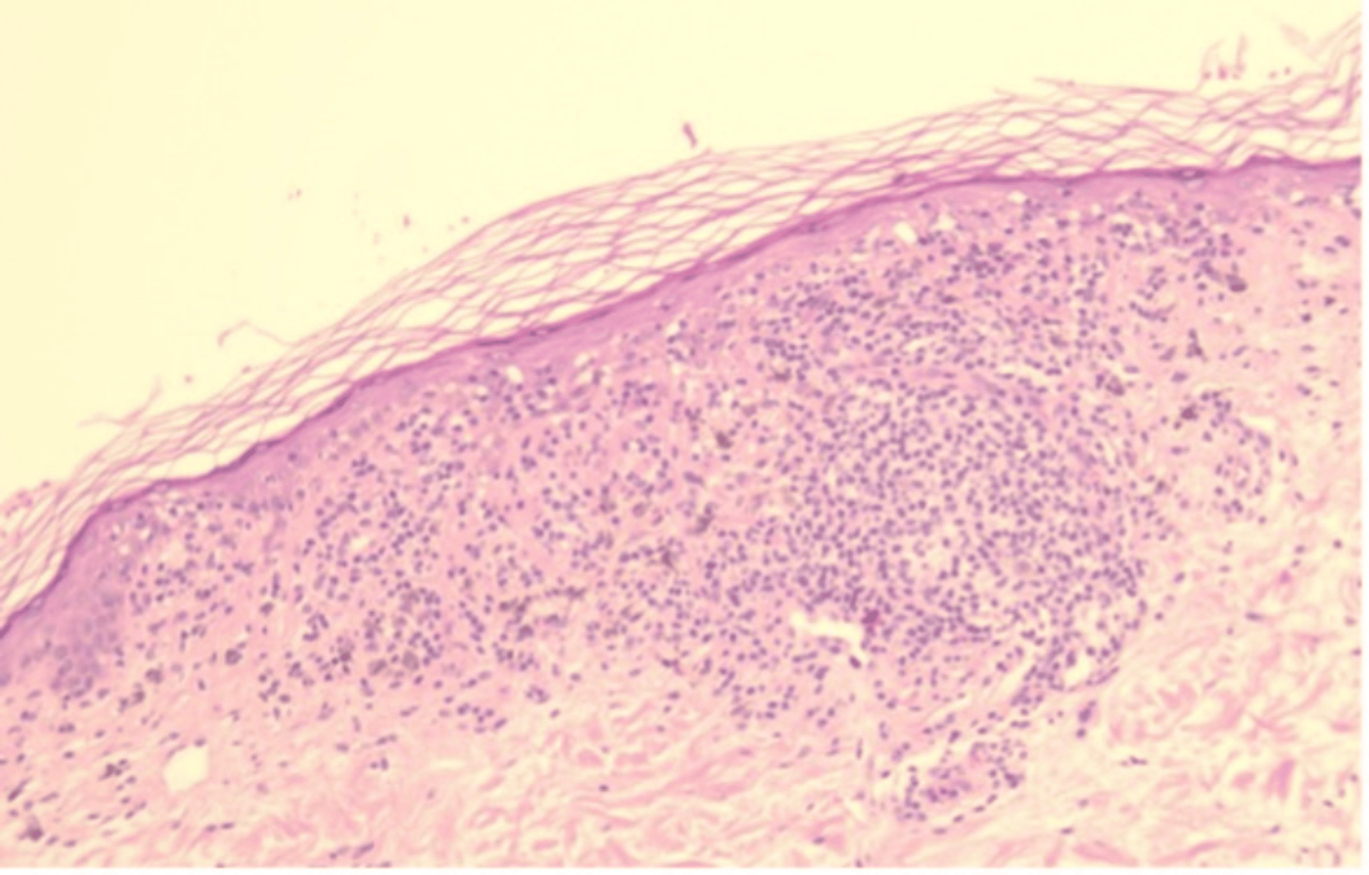
H&E stain, low power (×10): Epidermis showing compact hyperkeratosis, focal thinning, and a dense band-like lymphocytic infiltrate at the dermoepidermal junction H&E: hematoxylin and eosin

**Figure 3:**
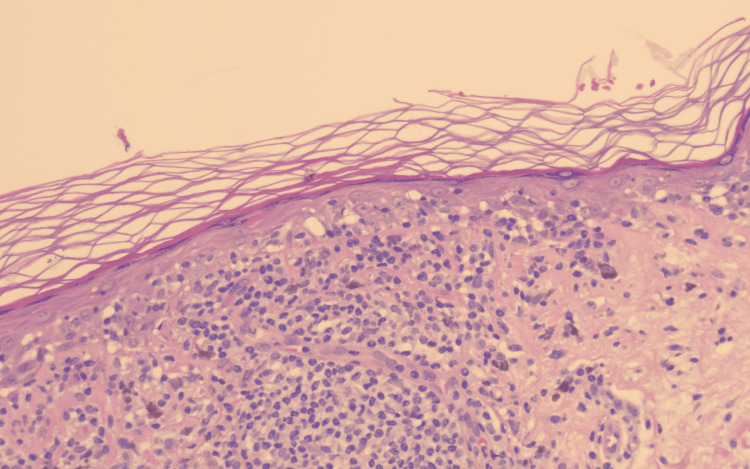
H&E stain, high power (×20): Basal vacuolar alteration with apoptotic keratinocytes (Civatte bodies) and pigment incontinence containing numerous dermal melanophages H&E: hematoxylin and eosin

Histopathologic examination revealed features consistent with a lichenoid interface dermatitis. The epidermis showed compact hyperkeratosis with focal thinning and areas of mild epidermal hyperplasia (Figure 2). There was basal cell vacuolar degeneration accompanied by a dense, band-like lymphocytic infiltrate at the dermoepidermal junction. Apoptotic keratinocytes (Civatte bodies) and pigment incontinence with numerous dermal melanophages were evident within the papillary dermis (Figure 3). No cytologic atypia, mitotic activity, or features of malignancy were identified. These findings were diagnostic of BLK.

Postoperative recovery was uneventful, and the wound healed with minimal scarring. At the three-month follow-up visit, no recurrence or new lesions were observed. The patient was reassured regarding the benign nature of the lesion and advised to attend routine dermatologic surveillance.

## Discussion

BLK is a regressive inflammatory lesion considered the endpoint of various epidermal proliferations, particularly solar lentigo and seborrheic keratosis [1,2]. The lesion arises through an interface dermatitis process in which keratinocytes undergo immune-mediated injury, producing a characteristic lichenoid infiltrate and pigmentary alteration [3]. Although BLK most commonly occurs on chronically sun-exposed areas, reports of BLK developing on non-sun-exposed regions such as the breast remain exceedingly rare [4]. Such atypical localization often raises strong clinical suspicion for melanoma or other neoplastic processes, highlighting the necessity of integrating clinical and histopathologic findings to establish an accurate diagnosis.

The pathogenesis of BLK is incompletely understood. UV radiation, epidermal aging, and immune-mediated mechanisms appear to act synergistically in initiating keratinocyte injury and promoting regression [2,5]. However, BLK can also occur in non-sun-exposed sites, suggesting additional triggers such as minor unnoticed trauma, localized immune responses, or intrinsic regression of pre-existing lesions independent of UV exposure. Histologic studies propose that BLK progresses through sequential inflammatory phases, beginning with an active lichenoid stage and evolving toward a late regressed phase characterized by prominent dermal melanophages and pigment incontinence [5,6].

Histopathologically, BLK demonstrates the classic features of a lichenoid interface dermatitis, including compact hyperkeratosis, basal cell vacuolar degeneration, apoptotic keratinocytes (Civatte bodies), and a dense, band-like lymphocytic infiltrate at the dermoepidermal junction [1,6]. Dermal melanophages and melanin incontinence reflect ongoing regression and are consistently reported across published cases. Epidermal changes may range from mild atrophy to irregular acanthosis depending on the stage of evolution [3,4]. In our case, compact hyperkeratosis, focal epidermal thinning, and mild epidermal hyperplasia were accompanied by basal cell vacuolar alteration and prominent pigment incontinence, findings well-aligned with the described histopathologic spectrum of BLK [1,5,7].

The differential diagnosis encompasses both lichenoid and melanocytic lesions. Lichen planus typically presents with multiple pruritic, violaceous papules and often involves mucosal surfaces, unlike the solitary, asymptomatic lesion seen in BLK. Seborrheic keratosis demonstrates papillomatosis and horn cysts histologically, features absent in BLK. Actinic keratosis exhibits keratinocytic atypia and parakeratosis, while melanoma is distinguished by disorganized nests of atypical melanocytes, pagetoid spread, and marked cytologic atypia [8]. On the breast, pigmented Paget disease is an additional important consideration due to its location and clinical pigmentation; however, it shows large atypical pagetoid cells infiltrating the epidermis, a pattern not seen in BLK. These distinctions underscore the indispensable role of histopathologic evaluation, particularly when BLK presents in unusual anatomic sites.

Emerging studies further support the use of dermoscopy and immunohistochemistry (IHC) in challenging cases. Dermoscopy often reveals gray dots, peppering, or granularity corresponding to melanophages, while IHC may demonstrate a predominance of CD8-positive lymphocytes in lichenoid infiltrates, helping differentiate regressive inflammatory lesions from melanocytic proliferations [7,8]. Incorporating these modalities may enhance diagnostic accuracy in ambiguous or atypical presentations.

Overall, the prognosis of BLK is excellent. Most lesions remain stable or regress spontaneously over time. Complete excision provides both diagnostic confirmation and definitive treatment, as demonstrated in our patient. Awareness of the variable clinical and histopathologic manifestations of BLK, including rare occurrences on non-sun-exposed areas such as the breast, can assist clinicians and pathologists in avoiding misdiagnosis, unnecessary wide local excisions, and patient distress.

## Conclusions

BLK is a regressive inflammatory lesion that can clinically and histologically mimic a variety of melanocytic and non-melanocytic conditions. This case highlights the importance of careful clinicopathologic correlation, particularly when lesions arise on atypical, non-sun-exposed sites such as the breast, where the suspicion for melanoma is often heightened. Recognition of BLK in this location is essential, as it should be included in the differential diagnosis of pigmented breast lesions to avoid unnecessary wide excisions or patient anxiety. Given its benign nature, complete excision is both diagnostic and therapeutic, and routine dermatologic follow-up is generally sufficient.
